# Neurogenic bladder in the multiple sclerosis population: A systematic review and meta-analysis of prevalence and a proposed evaluation algorithm

**DOI:** 10.1007/s10072-026-09289-6

**Published:** 2026-08-07

**Authors:** Vasileios Giannopapas, Evangelia Makrina Dimitriadou, Dimitrios K. Kitsos, Alexandra Akrivaki, Maria-Ioanna Stefanou, Konstantina Stavrogianni, Athanasios K. Chasiotis, John S. Tzartos, Daphne Bakalidou, Georgios Tsivgoulis, Georgios P. Paraskevas, Konstantinos Tsamis, Sotirios Giannopoulos

**Affiliations:** 1 Second Department of Neurology, School of Medicine, National and Kapodistrian University of Athens, Athens, Greece; 2 Interdisciplinary Laboratory of Research, Education and Disability Support (IREDS Lab), Department of Physiotherapy, Faculty of Health and Caring Professions, University of West Attica, Attica, Greece; 3 Department of Physiology, Faculty of Medicine, School of Health Sciences, University of Ioannina, Ioannina, Greece

**Keywords:** Multiple sclerosis, Neurogenic bladder, Lower urinary tract dysfunction, Prevalence, Meta-analysis

## Abstract

**Background:**

Neurogenic bladder (NGB) is one of the most common and debilitating manifestations of multiple sclerosis (MS), substantially impairing quality of life and increasing the risk of secondary complications.

**Objective:**

To determine the pooled prevalence of NGB and its subtypes in people with MS (PwMS).

**Methods:**

A systematic review and meta-analysis was conducted in accordance with PRISMA and MOOSE guidelines and was prospectively registered in PROSPERO. The MEDLINE, Embase, Scopus, and Google Scholar databases were searched. Risk of bias was assessed using the ROBINS-E scale. Pooled prevalence estimates were calculated using a random-intercept generalized linear mixed model (GLMM) with logit transformation and Hartung-Knapp adjustment. Meta-regression and subgroup analyses were performed to explore heterogeneity, and sensitivity analyses included leave-one-out analysis and prediction intervals.

**Results:**

Twenty-two studies comprising 39,304 PwMS were included. The pooled prevalence of NGB was 48.8% (95% CI: 34.1–63.7%). Subtype analyses showed pooled prevalences of 39.1% for storage symptoms, 21.4% for voiding symptoms, and 40.9% for mixed symptoms. Meta-regression did not identify significant associations between NGB prevalence and age, EDSS, or disease duration. Sensitivity and subgroup analyses indicated that heterogeneity (I² > 97%) was primarily attributable to differences in diagnostic definitions, assessment instruments, and study design rather than demographic or clinical characteristics.

**Conclusions:**

NGB affects approximately one in two PwMS with the prevalence ranging from 6 to 94%. Standardized screening and assessment strategies are warranted to improve early identification and optimize care pathways.

**Supplementary Information:**

The online version contains supplementary material available at https://doi.org/10.1007/s10072-026-09289-6.

## Introduction

Multiple sclerosis (MS) is a chronic, immune-mediated demyelinating disorder of the central nervous system characterized by neuroinflammation and neurodegeneration. People with MS (PwMS) experience a wide range of motor, sensory, cognitive, and autonomic symptoms [1]. Among these symptoms, neurogenic bladder (NGB) (also known as lower urinary tract dysfunction) is one of the most prevalent and debilitating dysfunction that significantly impairs functionality, daily activities, socialization, sexual function and lowers the quality of life of PwMS [2]. Neurogenic bladder in MS arises from demyelinating lesions that disrupt the neural pathways responsible for coordinated storage and voiding functions of the bladder [3]. Lesions involving the cerebral cortex result in loss of inhibitory control over the pontine micturition center (PMC) while lesions in the brainstem disrupt the coordination between detrusor and urethral sphincter a phenomenon known as detrusor-sphincter dyssynergia (DSD) that causes voiding dysfunction and incomplete bladder emptying [4]. Supra-sacral lesions in the Cervical and Thoracic spinal cord (CSS, TSS) interrupt descending inhibitory and facilitatory pathways leading to detrusor overactivity, elevated intravesical pressure, impaired bladder compliance and DSD [4, 5].

Clinically, these pathophysiological disturbances manifest as a broad spectrum of lower urinary tract symptoms, which are categorized into storage, voiding, and mixed symptoms. Storage symptoms include urinary urgency (with or without bladder spasms), increased urinary frequency, nocturia, and urinary incontinence, whereas voiding symptoms include urinary hesitancy, incomplete bladder emptying, and urinary retention [6].Given the heterogeneous and concurrent distribution of lesions, PwMS may experience a combination of the aforementioned symptoms while the majority of PwMS classify bladder dysfunction as one of the main factors affecting QoL with urinary incontinence being characterized as one of the “ worst” aspects of MS [7].

Regarding the physical implication of NGB on PwMS, recurrent urinary tract, bladder infections, bladder overdistention and pyelonephritis are some of the most common complications with recent studies also linking NGB to gait disturbances, increased fall risk and increased risk of frailty [8, 9].Beyond the physical domain, NGB negatively affects psychosocial aspects in the life of PwMS ranging from increased levels of fatigue, sleep disturbances, anxiety, depression, social anxiety and embarrassment as well as sexual function and sexual satisfaction [10].

In PwMS, lower urinary tract dysfunction has been associated with several risk factors including longer disease duration, higher neurological disability (EDSS score), older age, male sex, and spinal cord involvement, as well as objective markers of impaired bladder emptying (high post-void residuals and adverse urodynamic findings) [11]. Additionally, based on the literature review by De Sèze and colleagues (2007),longer disease duration, use of an indwelling catheter, high-amplitude neurogenic detrusor contractions, elevated detrusor pressures, presence of detrusor-sphincter dyssynergia, male sex, and age greater than 50 years increase the risk for upper urinary tract dysfunction [12]. NGB evaluation involves a combination of patient-reported outcome measures and objective urodynamic assessment. Self-report questionnaires, such as the Neurogenic Bladder Symptom Score and the Actionable Bladder Symptom Screening Tool, are commonly used for initial symptom assessment and follow-ups while post-void residual volume represents a first-line investigative tool. In patients with more severe or complex urinary symptoms, comprehensive urodynamic testing (including uroflowmetry, filling cystometry, and pressure-flow studies) is recommended [13–15].

Regarding the management of NGB, treatment predominantly focuses on symptomatic therapies and prevention of complications. Patients are typically prescribed anticholinergics and/or β3-adrenergic receptor agonists and intermittent catheterization in cases of DSD and/or increased post-void bladder residual [16]. Furthermore, recent studies have demonstrated the efficacy of physical therapy interventions (neuromodulation via tibial nerve stimulation, pelvic floor exercises) and of cannabinoids in the management of bladder and sexual dysfunction [17–19].

Despite the high prevalence and the negative impact of the patient’s QoL, NGB still remains underreported by patients (mainly due to stigma) and underrecognized by clinicians [20, 21].This systematic review and meta-analysis (SR-MA) aims to comprehensively evaluate the prevalence of NGB and NGB subtypes (storage, voiding and mixed symptoms) and examine potential relationships between the demographic and disease related characteristic and the presence of NGB in order to enhance understanding of the magnitude of NGB in PwMS.

## Methods

### Standard protocol approval-Registration

The pre-defined protocol for this systematic review and meta-analysis is registered in the Prospero database (CRD420251246360). This systematic review and meta-analysis adhere to the Preferred Reporting Items for Systematic Reviews and Meta-Analysis (PRISMA) and was written in accordance to the Meta-Analysis of Observational Studies in Epidemiology proposal. Due to the nature of this study (review) no ethics board approval or written informed consent was required.

### Data sources-study selection

The systematic literature search was executed on January 10th 2026 and included two major medical databases, MedLine PubMed and Embase, Scopus as well as the first 200 results of Google Scholar (shorted by relevance). Two independent reviewers (VG, EMD) searched the databases for relevant records using the following terms: “multiple sclerosis”, “neurogenic bladder”, “bladder dysfunction”, “voiding dysfunction”,” urinary incontinence”. The PECO strategy and complete search algorithms used in MEDLINE and Scopus and EMBASE are provided in the Supplementary materials. Population-based studies, MS registries, and observational epidemiologic designs-including cross-sectional, cohort, and registry-based studies-reporting on neurogenic bladder (NGB) or lower urinary tract dysfunction (LUTD) in people with multiple sclerosis (PwMS) were considered eligible for inclusion. Eligible studies involved adults aged 18 years or older diagnosed with MS according to the 2017 revised McDonald criteria or earlier accepted McDonald criteria for studies conducted prior to 2017. Inclusion further required that studies report, or allow calculation of, prevalence or frequency estimates of NGB in PwMS and employ systematic, consecutive, or registry-based patient identification.

In accordance with the predefined protocol, studies were excluded if they enrolled participants younger than 18 years; focused on non-MS neurological populations without separately reported MS-specific urinary outcomes; lacked extractable urinary data attributable to MS; or did not report or permit calculation of NGB prevalence. Interventional or experimental trials—including drug-specific or treatment-focused studies in which urinary outcomes were incidental and not systematically collected-were excluded, as were studies relying on purposive or convenience sampling unless conducted within a clearly representative MS registry. Studies with sample sizes below 30, consistent with methodological expectations for stable prevalence estimation, and publication types such as case reports, case series, reviews, commentaries, letters, dissertations, pre-prints, and conference abstracts were also excluded. Potentially eligible studies were required to have recruited participants through systematic or consecutive patient identification within defined clinical or registry settings; studies relying on self-selected, volunteer, or web-based recruitment were excluded regardless of sample size.

When multiple publications drew on overlapping cohorts, the most complete or most recent dataset was retained. All included studies were required to be published in English between 1 January 2010 and 31 December 2025, and all retrieved studies underwent independent assessment by two reviewers (VG, EMD) and any disagreements were resolved after discussion with the senior author (SG).

### Quality control, bias assessment and data extraction

The risk of bias for relevant domains in each included study was assessed using the Risk Of Bias In Non-randomized Studies of Exposure (ROBINS-E) tool [22]. Quality control and bias assessment was conducted independently by two reviewers (VG, EMD), and disagreements were settled by consensus after discussion with the senior author (SG). Data from individual studies, including author names, date of publication, study design, country, event type, and patient characteristics, were extracted in structured reports for further analyses. Additionally, the JBI Critical Appraisal Checklist for Studies Reporting Prevalence Data was assessed [23].

### Outcomes

An aggregate data meta-analysis was performed with the inclusion of the identified population-based studies or registries, and observational cohort studies.

The predefined primary outcome measures were twofold: (i) the pooled prevalence of NGB in PwMS; and (ii) the pooled prevalence of NGB subtypes (voiding, storage, mixed) in PwMS. Secondary outcomes of interest comprised potential associations between demographic and MS-related characteristics and NGB in the MS population.

For the purpsoses of this study NGB was defined as “lower urinary tract dysfunction attributable to a neurological disorder, encompassing storage symptoms (urgency, frequency, nocturia, incontinence), voiding symptoms (hesitancy, incomplete emptying, retention), and mixed presentations”.

### Statistical analysis

For the aggregate meta-analysis, a random intercept generalized linear mixed model (GLMM) with a logit link was used to estimate pooled prevalence values and corresponding 95% confidence intervals. This modeling framework directly accounts for the binomial distribution of proportion data and provides stable variance estimation, particularly when the rates of events approach the extremes of 0 or 1 or when study sample sizes vary substantially [24]. Between-study variance was estimated using maximum likelihood, and confidence intervals were calculated using the Hartung-Knapp adjustment to enhance inferential reliability in the presence of substantial heterogeneity in all primary and subtype GLMM models [25]. A random-effects model employing a logit transformation (without Hartung-Knapp adjustment) was subsequently fitted to evaluate the robustness of pooled estimates to alternative variance-stabilizing approaches. A 95% prediction interval was derived from the GLMM to estimate the range of true prevalence [22]. Additionally, prespecified exploratory analysis based on NGB symptomatology were conducted to estimate the pooled prevalence of storage, voiding, and mixed symptoms in PwMS but given the small number of contributing studies the resulting estimates should be interpreted with caution.

To explore sources of heterogeneity and determine whether study-level factors were associated with prevalence estimates, univariate and multivariable meta-regression analyses were conducted with the following features, age, Expanded Disability Status Scale (EDSS) score, and disease duration, entered as moderators [25]. Quadratic terms were employed to assess potential non-linear associations, and a hierarchical meta-regression framework was used to evaluate the incremental contribution of each moderator, beginning with EDSS, followed by sequential addition of age and disease duration [26]. Restricted maximum-likelihood estimation was used for all models, and relative model fit was compared using the Akaike Information Criterion and pseudo-R² values relative to the intercept-only model [25].

A sensitivity analysis stratified by study design was performed to examine whether methodological characteristics influenced prevalence estimates. Study influence and model robustness were further assessed through leave-one-out analyses, in which the GLMM was iteratively refitted after exclusion of each study [27]. Publication bias and small-study effects were evaluated through visual inspection of funnel plots, statistical testing using linear regression methods, and implementation of trim-and-fill procedures when appropriate [28, 29]. All statistical analyses were conducted using R (R Studio for ioS (RStudio 2023.12.1 + 402 (“Ocean Storm”)), employing the r-meta package via “metaprop ()” for pooled prevalences and the “metareg()” for meta-regressions, with two-tailed significance set at α = 0.05.

### Data availability

All data generated or analyzed during this study are included in the main text and the Supplementary Materials. The raw datasets are available from the corresponding author upon reasonable request.

## Results

### Literature search

**Fig. 1:**
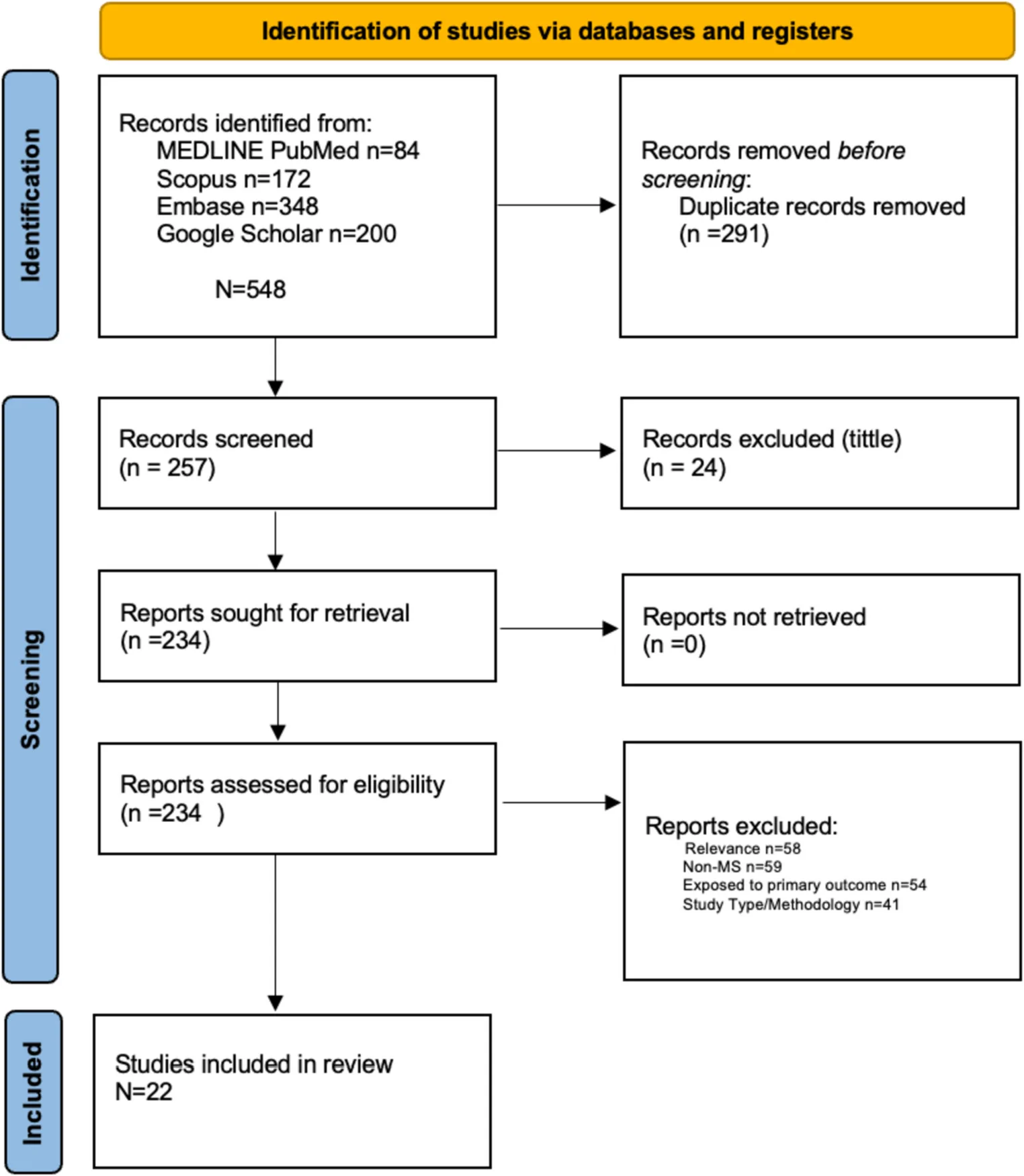
PRISMA Flowchart

The systematic literature search yielded 548 results. After the exclusion of 291 duplicate records and the pre-screening of the records based on title and abstract 234 records were assessed. Based on the pre-defined inclusion-exclusion criteria 22 records were eligible for inclusion (Fig. 1).

### Bias assessment

Eligible articles underwent bias assessment using the ROBINS-E scale. There was a low to moderate degree of bias mainly due to measurement and reporting of outcomes (Supplementary Fig. 1). JBI Critical Appraisal Checklist assessment was consistent with the ROBINS-E findings, demonstrating low-to-moderate risk of bias across all 22 included studies, with moderate risk confined primarily to the Missing Data and Outcome Measurement domains (Supplementary Table 1).

### Primary outcomes

**Fig. 2:**
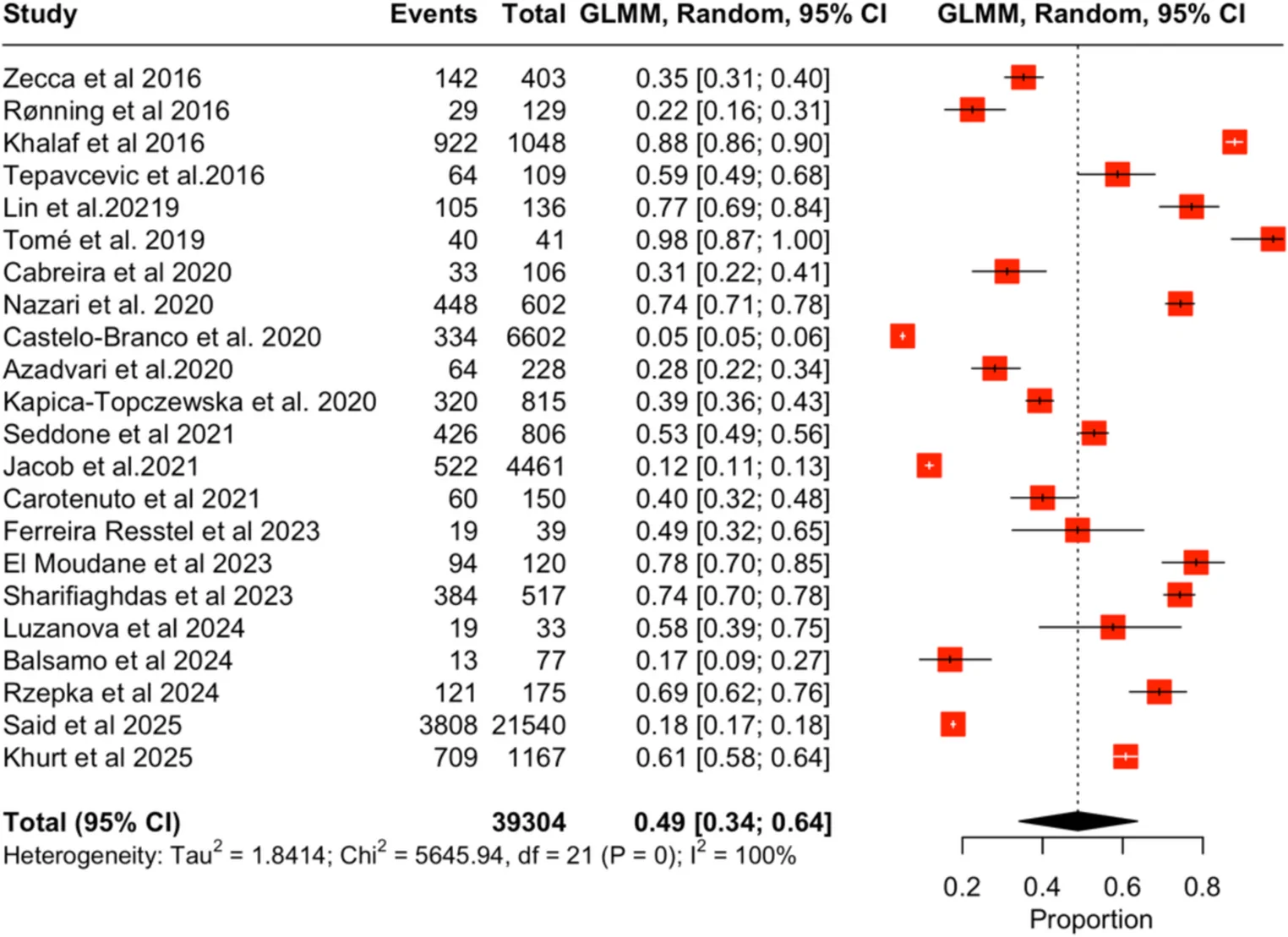
Pooled prevalence of NGB in PwMS

**Fig. 3:**
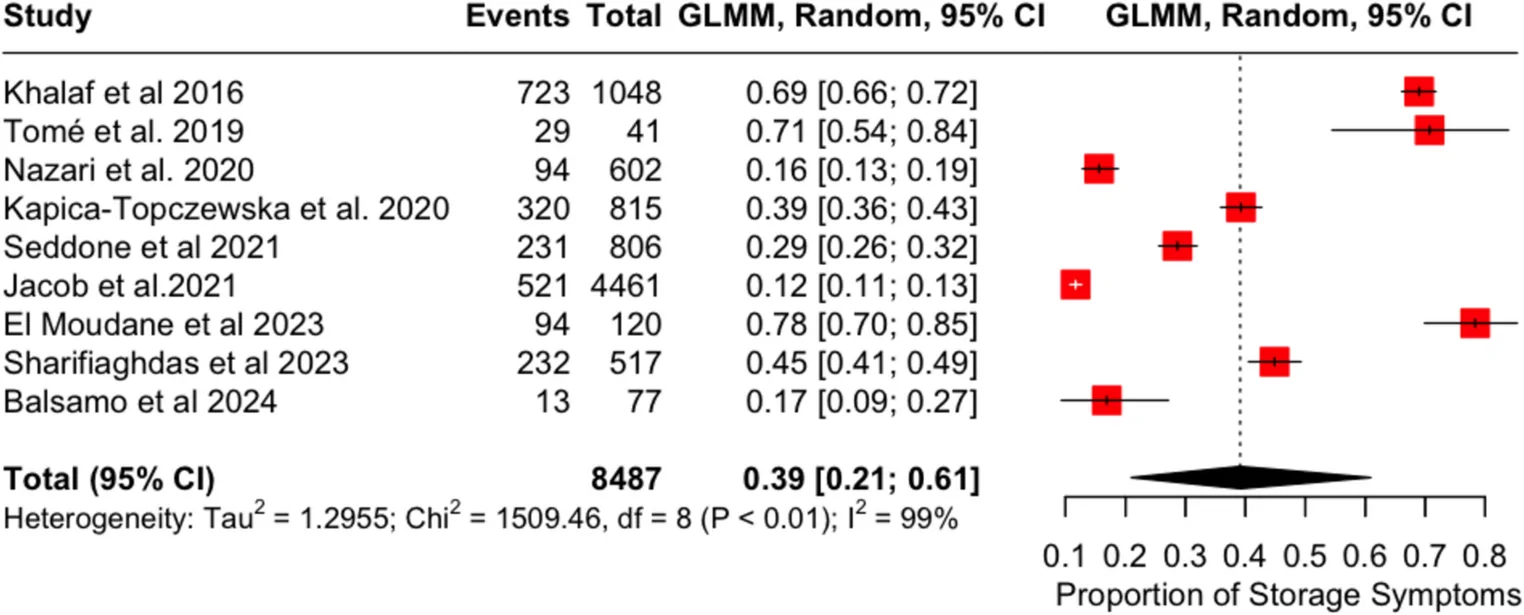
Pooled prevalence of Storage Symptoms in PwMS

**Fig. 4:**
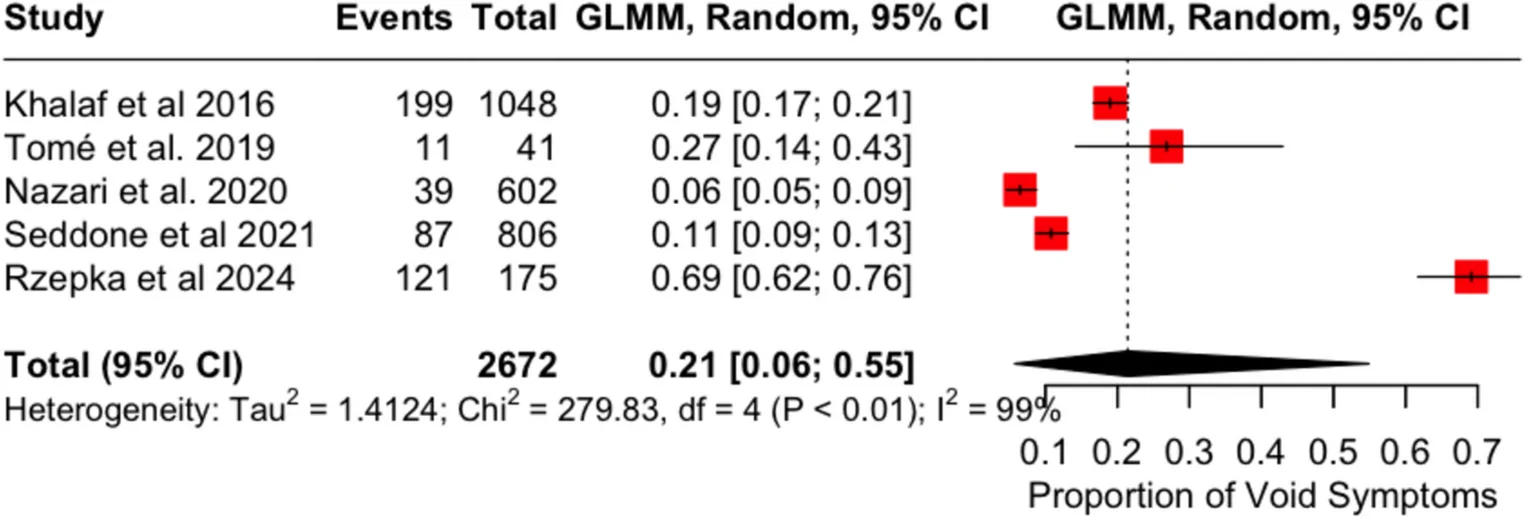
Pooled prevalence of voiding symptoms in PwMS

**Fig. 5:**
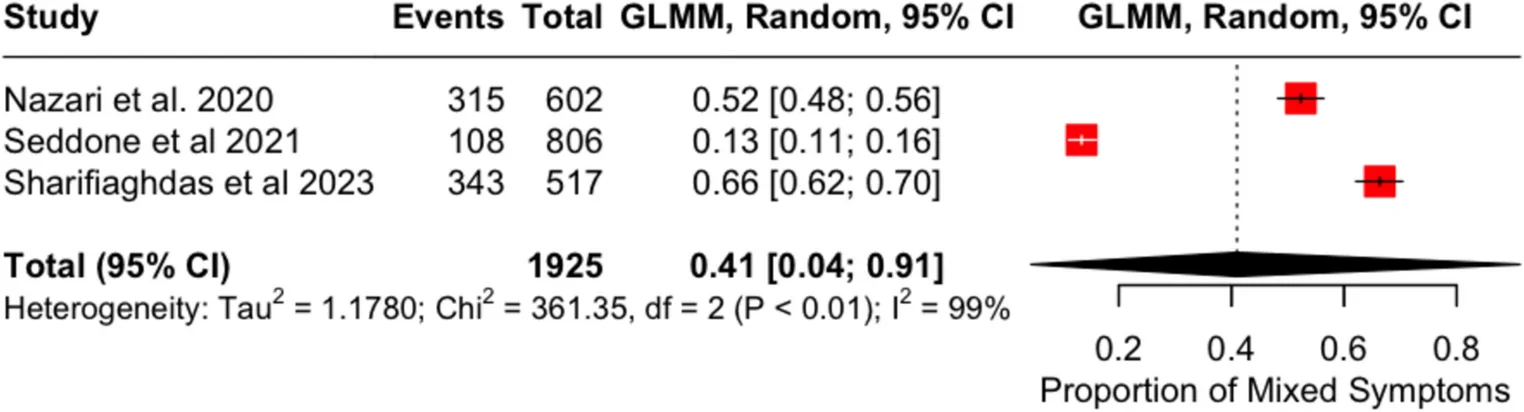
Pooled prevalence of mixed neurogenic bladder symptoms in PwMS

The primary meta-analysis included 22 studies [20, 21, 27, 29–48] comprising 39,304 participants, of whom 8,676 had NGB symptoms (Table 1). Using a random-intercept logistic regression model (GLMM), the pooled prevalence was 48.8% (95% CI: 34.1% to 63.7%) (Fig. 2). Between-study heterogeneity was extremely high (τ² = 1.84; I² = 99.6%), indicating substantial variability in prevalence estimates across studies. Both the Wald and likelihood ratio heterogeneity tests were highly significant (*p* < 0.001), confirming the presence of real differences between studies beyond chance. Subsequent meta-analyses of proportion were performed to produce the pooled prevalence for each NGB subtype. Storage symptoms had a pooled prevalence of 39.1% (95% CI: 20.9% to 60.9%) (Fig. 3) with between-study heterogeneity was extremely high (τ² = 1.29; I² = 99.5%), voiding symptoms had a pooled prevalence of 21.4% (95% CI: 5.7% to 54.9%)(τ² = 1.41; I² = 99.8%) (Fig. 4) and mixed symptoms had a pooled prevalence of 40.9% (95% CI: 4.4% to 91.2) (τ² = 1.17; I² = 99.4%) (Fig. 5).

**Table 1:** Included studies characteristics

Author (Year)	Region	Type	*N*	Age	EDSS	Disease Duration	NGB
Zecca et al. (2016) [30]	Switzerland	CS	403	44.3	3.1	11.8	142
Rønning et al. (2016) [31]	Norway	Observational	129	56	7.5	33.4	29
Khalaf et al. (2016) [32]	USA	Observational	1048	47.8		8.5	922
Tepavcevic et al. (2016) [33]	Serbia	CS	109	41.5	4.2	9.2	64
Lin et al. (2019) [34]	Australia	Observational	136	56.1		18.2	105
Tomé et al. (2019) [35]	Brazil	CS	41	40.7	3	10.5	40
Cabreira et al. (2020) [36]	Portugal	Cohort	106		4.2		33
Nazari et al. (2020) [37]	Iran	CS	602	35.4	2.45	7.6	448
Castelo-Branco et al. (2020) [21]	Sweden	CS	6602	40.9			334
Azadvari et al. (2020) [38]	Iran	CS	228		3.6	2.6	64
Kapica-Topczewska et al. (2020) [39]	Austria	Cohort	815	44.7	3.3		320
Seddone et al. (2021) [40]	Italy	CS	806	45.8	4	12.5	426
Jacob et al. (2021) [41]	Germany	Cohort	4461	44.2			522
Carotenuto et al. (2021) [42]	Italy	Observational	150	43.3	3.5	11.8	60
Ferreira Resstel et al. (2023) [43]	Brazil	Observational	39				19
El Moudane et al. (2023) [44]	Morocco	CS	120	43			94
Sharifiaghdas et al. (2023) [45]	Iran	CS	517	37.9		27.7	384
Luzanova et al. (2024) [46]	Russia	Observational	33	36	3.5	13	19
Balsamo et al. (2024) [47]	Italy	Observational	77	36.2	4	13.1	13
Rzepka et al. (2024) [48]	Poland	CS	175	38.8	2.4	7.4	121
Said et al. (2025) [20]	USA	Cohort	21,540	44.9			3808
Krhut et al. (2025) [27]	Czech Republic	Cohort	1167	45		11	709

Age (years), Disease Duration (years), EDSS, all variable are presented in mean, CS: Cross-Sectional

### Secondary outcomes

Meta-regression analyses were conducted to examine whether Age, disability level (EDSS), and disease duration accounted for the substantial heterogeneity observed in the prevalence of NGB across studies. In univariate models, none of the moderators demonstrated a significant association with prevalence, with Age (*p* = 0.862), EDSS (*p* = 0.369), and Disease Duration (*p* = 0.532) all showing small, non-significant effects. The multivariate model, which included all three predictors simultaneously, also failed to identify any statistically significant relationships (moderator: F [3, 7] = 1.88, *p* = 0.221). Although disease duration showed a relatively larger effect size in the multivariate model (β = −0.1092), this trend remained non-significant. Across all models, residual heterogeneity was high (I² > 97%), indicating a low degree of explainability. The cumulative results of these analysis indicate that variations in age distribution, disability severity, and disease duration across studies do not meaningfully contribute to between-study differences and that heterogeneity is likely due to methodological and contextual factors (study design, diagnostic definitions, population selection, and measurement).

A hierarchical (sequential) multivariable meta-regression was also performed to evaluate the incremental contribution of each moderator. None of the individual moderators were statistically significant, but the hierarchical meta-regression incorporating all three variables indicated that the combination of EDSS, age, and disease duration explained a substantial proportion of between-study heterogeneity (pseudo-R² = 64.3%) with age proving the largest incremental improvement, followed by disease duration. This finding should be interpreted with caution given that the model was based on only 11 studies with complete covariate data and may reflect a reduction in estimated between-study variance rather than a statistically confirmed causal explanation.

Subgroup analyses based on study design showed notable variation in the estimated prevalence of NGB among PwMS, although differences did not reach statistical significance. Cross-sectional studies reported the highest pooled proportion (0.58; 95% CI: 0.31–0.81), followed by observational studies (0.51; 95% CI: 0.25–0.76), whereas cohort studies showed a substantially lower prevalence (0.29; 95% CI: 0.12–0.55).Despite these differences, the test for subgroup effects was not significant (Q = 4.38, df = 2, *p* = 0.11).

Leave-one-out sensitivity analysis demonstrated that exclusion of any single study resulted in minimal changes in the pooled estimate. The logit-transformed pooled proportion varied only between − 0.19 and 0.09 across the 22 refitted models, corresponding to an absolute prevalence shift of less than ± 4% which indicates that no individual study exerted undue influence on the meta-analytic result and that the overall pooled prevalence is robust (Supplementary Table 2).

Finally, A 95% prediction interval was computed to estimate the range of true prevalence values expected in future similar studies. The prediction interval was very wide, ranging from 5.9% to 93.5% on the proportion scale, reflecting the extremely high between-study heterogeneity (I² > 97%). This interval indicates that while the pooled estimate provides an overall summary, individual studies may report substantially different prevalence values depending on population characteristics, diagnostic methods, and study design (Supplementary Table 3).

Taken together, the results of the subgroup, leave-one -out and prediction interval analyses indicate that the substantial heterogeneity is a more likely consequence of the variability in diagnostic criteria, population characteristics, or outcome definitions likely contribute more to heterogeneity than sample size and study design.

### Publication bias

The funnel plot of logit-transformed proportions demonstrated noticeable asymmetry, largely driven by substantial heterogeneity and the presence of several small studies reporting extreme prevalence estimates. The distribution of small studies was uneven, suggesting possible small-study effects; however, given the very high heterogeneity (I² > 97%), the asymmetry likely reflects genuine between-study differences rather than publication bias alone. Larger studies showed more consistent, centrally clustered estimates, supporting the stability of the overall pooled effect (Supplementary Fig. 2). Subsequent exploratory analyses regarding publication bias were performed. Egger’s test suggested significant funnel plot asymmetry (t = 2.78, *p* = 0.012), with a substantial bias coefficient. The trim-and-fill method imputed 10 additional studies, yielding an adjusted pooled prevalence of 0.21 (95% CI: 0.10–0.38); nevertheless, suggesting lower prevalence when adjusting for symmetry but the corrected model retained extreme heterogeneity (I² = 99.7%), that indicates genuine clinical and methodological variability across studies rather than selective publication, limiting the interpretability of this adjustment. Overall, observed funnel asymmetry appears attributable to true study variability rather than selective reporting (Supplementary Table 4).

## Discussion

The present SR-MA found that neurogenic bladder is highly prevalent among individuals with multiple sclerosis, with a pooled prevalence of 48.8% (95% CI: 34.1%−63.7%) across more than 39,304 PwMS. Subtype-specific analyses showed similarly elevated estimates, with storage symptoms occurring in 39.1% (95% CI: 20.9%−60.9%), voiding symptoms in 21.4% (95% CI: 5.7%−54.9%), and mixed symptoms in 40.9% (95% CI: 4.4%−91.2%) of PwMS, underscoring the complex and multifactorial nature of bladder dysfunction in MS. Although substantial heterogeneity was observed, meta-regression analyses indicated that demographic and disease related characteristics (age, disability level, and disease duration) did not significantly account for the variability in prevalence estimates, suggesting that methodological and contextual differences across studies are the more likely contributors. More specifically, leave-one-out analyses showed that no individual study materially influenced the pooled estimate, affirming the robustness of the overall finding.

The results of this SR-MA are in alignment with previous studies exploring the prevalence of bladder symptomatology in PwMS. In a recent SR-MA, Ghasemi and colleagues reported a 41% pooled prevalence of urge urinary incontinence and a 21% of stress urinary incontinence across 15.052 PwMS [49]. It is worth noting that urgency and stress urinary incontinence reflect only storage-phase abnormalities, and do not reflect the broader neurogenic bladder profile in MS.

NGB symptomatology exerts a wide-reaching influence on health, well-being and QoL of PwMS extending well beyond urinary function alone. Falls, occurring in approximately 40% of PwMS [50], have been linked to NGB through urinary urgency, nocturia, and rushing-to-void behaviour, with recent evidence demonstrating greater gait impairment, reduced dual-task performance, and amplified fear of falling in PwMS with NGB [9, 51–53]. Sexual dysfunction, another common and debilitating symptom of MS, has been linked to NGB by Kessler and colleagues as one of its most common secondary causes [10], with NGB symptomatology associated with moderate-to-severe erectile dysfunction and more severe sexual dysfunction in women with MS [54]; a recent study by Bientinesi and colleagues further highlighted that the co-occurrence of NGB and sexual dysfunction carries a significant negative impact on relationship satisfaction and QoL [55]. This interaction arises from shared neuroanatomical and neurophysiological pathways and MS-related autonomic dysregulation affecting both urinary and sexual function [55, 56]. Beyond physical consequences, NGB disrupts daily activities, socialization, and travel, generating chronic psychological stress and loss of environmental security, with PwMS attributing this effect to the unpredictability and variability in intensity of bladder symptomatology [8]. Finally, physical fatigue, one of the most prevalent symptoms in PwMS, has been identified as a statistically significant predictor of urinary quality of life, with a moderate positive correlation reported between fatigue-related symptomatology and urinary quality of life outcomes [57].

Taken together, these findings underscore that the high prevalence of NGB in PwMS, irrespective of the heterogeneity in how it is measured, has consistent and wide-reaching consequences across physical, psychological, and social domains. Standardisation of assessment methods, as reflected in the algorithm proposed below, is therefore not merely a methodological concern but a clinical imperative for ensuring that the burden of NGB is accurately captured and appropriately managed.

**Fig. 6:**
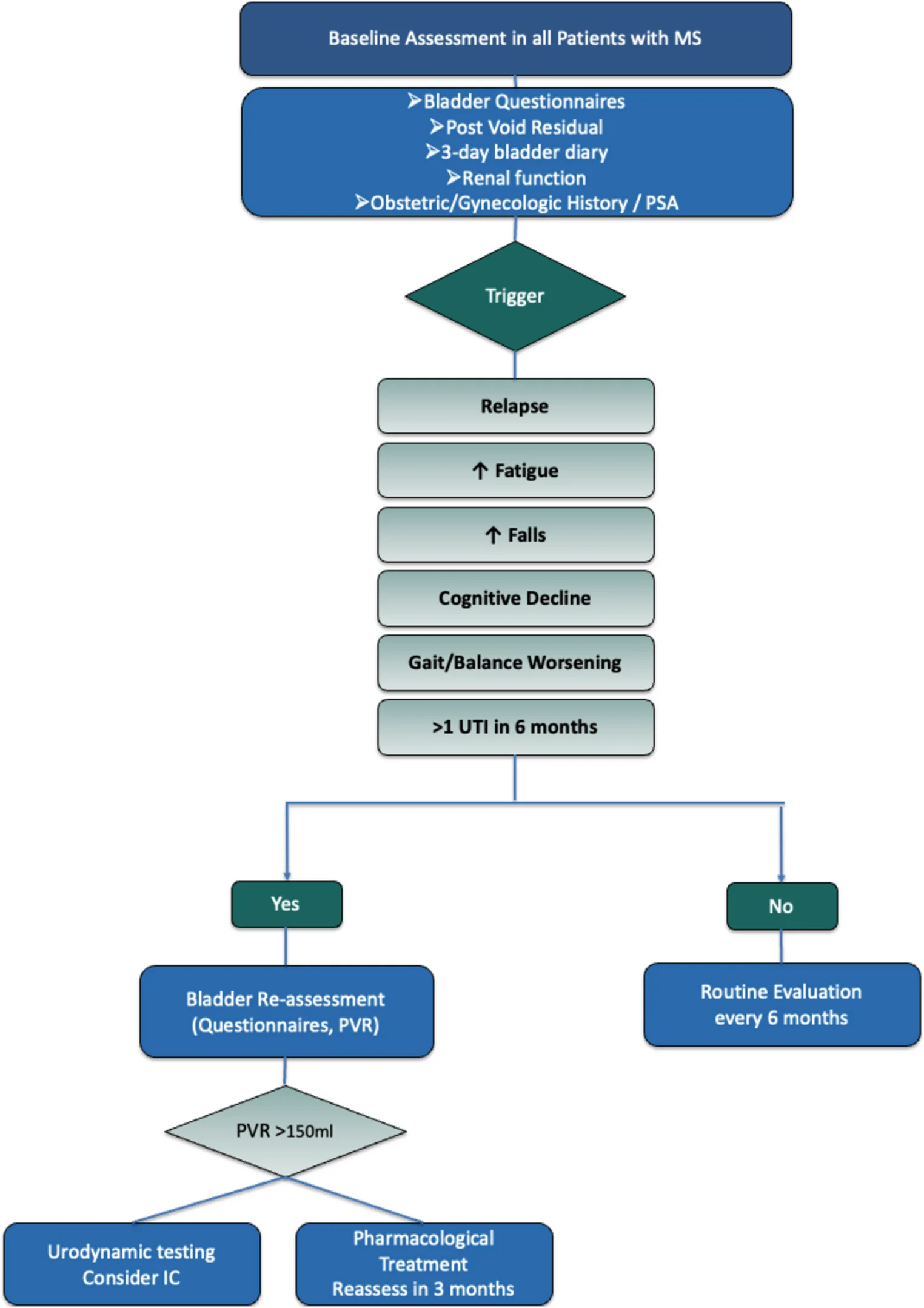
Proposed algorithm for the assessment of NGB in PwMS

Given the high prevalence of neurogenic bladder (NGB) in people with multiple sclerosis (PwMS) and the substantial heterogeneity across published studies-largely attributable to methodological and contextual differences in assessment strategies-we propose a standardized, neurologist-led screening and evaluation algorithm for the assessment of NGB in PwMS (Fig. 6). In accordance with the European Academy of Neurology/European Federation of Autonomic Societies/International Neuro-Urology Society (EAN/EFAS/INUS) NEUROGED guidelines, baseline evaluation includes a bladder symptom questionnaire, post-void residual (PVR) measurement (given that a PVR > 100 mL is the commonly accepted threshold for clinically significant incomplete emptying per EAN/EFAS/INUS guidelines), a 3-day bladder diary, and renal function assessment, with routine follow-up every six months as recommended by the NEUROGED guidelines [58]. Because urinary symptoms may fluctuate with disease activity and non-motor manifestations (specifically fatigue and cognitive decline), targeted reassessment is recommended following MS relapse, patient-reported symptoms change, or recurrent urinary tract infections (UTI) (> 1 in a 6 month period) [42, 57, 59]. Clinically meaningful symptom worsening should be performed using disease-specific instruments, including the Actionable Bladder Symptom Screening Tool, the Neurogenic Bladder Symptom Score, and the SF-Qualiveen [14, 15, 60] while objective reassessment should include PVR measurement, with moderate residual volumes managed initially by neurologist-led pharmacological treatment and higher PVR values or refractory symptoms triggering referral for specialist neuro-urological evaluation, including urodynamic testing, This stepwise algorithm aims to standardize NGB assessment in PwMS, support early identification of clinically relevant dysfunction, and optimize multidisciplinary care pathways, prioritizing non-invasive management techniques first (as stated in the NEUROGED recommendations). The proposed algorithm represents a clinical expert proposal informed by the EAN/EFAS/INUS NEUROGED guidelines by Panicker and colleagues 2025 and the findings of the current meta-analysis regarding the high prevalence and heterogeneity of NGB assessment in PwMS. It is not derived directly from the pooled prevalence estimate and should not be interpreted as a meta-analytically validated clinical pathway.

This study is not without limitations. First, there was a substantial degree of heterogeneity that is primarily attributed to methodological differences, differences in diagnostic criteria, assessment instruments and study design. Second, the majority of included studies relied on self-reporting symptoms rather than objective assessments of NGB (urodynamic testing, post void residual volume). Finally, cross-sectional designs predominated, limiting causal inferences regarding the relationship between NGB and clinical outcomes.

## Conclusion

Approximately one in two PwMS experience NGB symptomatology with the prevalence ranging (across varied clinical settings and assessment methods), from as low as 6% to as high as 94%.The complex and multifactorial nature of NGB substantially impacts patient’s lives, contributing to increased fall risk, fatigue, sexual dysfunction and psychological distress. Given the high frequency and the inherent heterogeneity in assessment methods a standardized screening algorithm is essential to ensure early detection and accurate recording of NGB findings.

## Supplementary Information

Below is the link to the electronic supplementary material.

Supplementary Material 1 (DOCX 1.75 MB)
