# Supplementary material for: Neurogenic bladder in the multiple sclerosis population: A systematic review and meta-analysis of prevalence and a proposed evaluation algorithm
Source: Neurol Sci. 2026 Aug 7;47(9):687. doi: 10.1007/s10072-026-09289-6 (PMC13447475; doi:10.1007/s10072-026-09289-6)
Supplement: Supplementary file 1 [file 10072_2026_9289_MOESM1_ESM.docx]

**Neurogenic bladder in the Multiple Sclerosis population: A systematic review and meta-analysis of prevalence and a proposed evaluation algorithm - Supplementary Materials**

**Contents:**

**1.PECO Strategy**

**2.Search Algorithm**

**3.Inclusion- Exclusion Criteria**

**4.Supplementary Tables**

1. Supplementary table 1: Leave one out analysis (GLMM)
2. Supplementary table 2: Prediction interval analysis
3. Supplementary table 3: Trim and Fill results

**5.Supplementary figures**

1. Supplementary figure 1: Robins-E traffic light plot
2. Supplementary figure 2: Funnel plot

**PICO Strategy**

|  | **Description** | **Rationale / Examples** |
| --- | --- | --- |
| **Population** | Adults (≥18 years) with a definite diagnosis of multiple sclerosis (MS) according to the 2017 revised McDonald criteria (or earlier McDonald versions if pre-2017). | Ensures diagnostic accuracy and excludes pediatric MS. |
| **Exposure** | Presence or reporting of neurogenic bladder (NGB) or lower urinary tract dysfunction (LUTD) associated with MS -identified clinically, urodynamically, or via patient-reported outcomes. | Captures urinary dysfunction whether it is the primary focus or reported incidentally (as a comorbidity, demographic, or baseline characteristic). |
| **Comparison** | Not applicable for prevalence studies; where available, comparisons may be made between MS patients with vs. without NGB. | Secondary analytic stratification. |
| **Outcome** | Prevalence or frequency of neurogenic bladder / LUTD in people with MS. Secondary data may include type of dysfunction (storage, voiding, mixed) or associations with disease subtype/duration. | Quantitative outcome needed for meta-analysis. |
| **Study design** | Observational studies (cross-sectional, cohort, registry, or epidemiologic designs) reporting prevalence or frequency data, including studies where NGB is described as a secondary variable or baseline characteristic. | Provides robust prevalence data for pooling; allows inclusion of studies that incidentally report NGB prevalence. |

**Search Algorithm**

| **Database** | **Search Period** | **Search Strategy / Syntax** |
| --- | --- | --- |
| **PubMed (MEDLINE)** | 1 Jan 2010 – 31 Dec 2025 | ( “Multiple Sclerosis”[Mesh] OR “multiple sclerosis”[tiab] )  AND ( “Urinary Bladder, Neurogenic”[Mesh] OR “neurogenic bladder”[tiab] OR “neurogenic lower urinary tract dysfunction”[tiab] OR “bladder dysfunction”[tiab] OR “urinary dysfunction”[tiab] OR “urinary symptoms”[tiab] OR “lower urinary tract symptoms”[tiab] OR LUTS[tiab] OR “voiding dysfunction”[tiab] OR “urinary incontinence”[tiab] OR “urinary retention”[tiab] )  AND ( “Prevalence”[Mesh] OR “Epidemiology”[Mesh] OR prevalence[tiab] OR incidence[tiab] OR frequency[tiab] OR characteristic*[tiab] OR comorbidit*[tiab] OR “clinical feature*”[tiab] OR “demographic*”[tiab] OR “baseline data”[tiab] OR “patient profile”[tiab] OR “disease feature*”[tiab] )  AND ( “Cross-Sectional Studies”[Mesh] OR “Cohort Studies”[Mesh] OR “Epidemiologic Studies”[Mesh] OR “Observational Study”[Publication Type] OR “Multicenter Study”[Publication Type] )  AND (“Humans”[Mesh])  AND (“2010/01/01”[Date - Publication] : “2025/12/31”[Date - Publication])  AND (english[lang]) |
| **Embase (Elsevier)** | 1 Jan 2010 – 31 Dec 2025 | (‘multiple sclerosis’/exp OR ‘multiple sclerosis’:ab,ti)  AND (‘neurogenic bladder’/exp OR ‘neurogenic bladder’:ab,ti OR ‘neurogenic lower urinary tract dysfunction’:ab,ti OR ‘bladder dysfunction’:ab,ti OR ‘urinary dysfunction’:ab,ti OR ‘urinary symptoms’:ab,ti OR ‘lower urinary tract symptoms’:ab,ti OR LUTS:ab,ti OR ‘voiding dysfunction’:ab,ti OR ‘urinary incontinence’:ab,ti OR ‘urinary retention’:ab,ti)  AND (‘prevalence’/exp OR ‘epidemiology’/exp OR prevalence:ab,ti OR incidence:ab,ti OR frequency:ab,ti OR characteristic*:ab,ti OR comorbidit*:ab,ti OR ‘clinical feature*’:ab,ti OR ‘demographic*’:ab,ti OR ‘baseline data’:ab,ti OR ‘patient profile’:ab,ti OR ‘disease feature*’:ab,ti)  AND (‘cross-sectional study’/exp OR ‘cohort study’/exp OR ‘observational study’/exp OR ‘epidemiologic study’/exp)  AND [humans]/lim  AND [english]/lim  AND [2010-2025]/py  AND [article]/lim |
| **Scopus (Elsevier)** | 1 Jan 2010 – 31 Dec 2025 | TITLE-ABS-KEY(“multiple sclerosis”)  AND TITLE-ABS-KEY(“neurogenic bladder” OR “neurogenic lower urinary tract dysfunction” OR “bladder dysfunction” OR “urinary dysfunction” OR “urinary symptoms” OR “lower urinary tract symptoms” OR LUTS OR “voiding dysfunction” OR “urinary retention” OR “urinary incontinence”)  AND TITLE-ABS-KEY(prevalence OR epidemiology OR incidence OR frequency OR characteristic* OR comorbidit* OR “clinical feature*” OR “demographic*” OR “baseline data” OR “patient profile” OR “disease feature*”)  AND TITLE-ABS-KEY(“cross-sectional” OR “cross sectional” OR cohort OR observational OR epidemiologic OR registry)  AND PUBYEAR > 2009 AND PUBYEAR < 2026  AND DOCTYPE(ar)  AND NOT TITLE-ABS-KEY(mouse OR mice OR rat OR “animal model” OR “in vitro”) |

Search algorithm verified on 12 November 2025; official database searches executed on 10 January 2026

**Inclusion and Exclusion Criteria**

| Inclusion Criteria | Exclusion Criteria |
| --- | --- |
| Adults (≥18 years) diagnosed with MS according to the 2017 revised McDonald criteria or earlier accepted criteria | Participants younger than 18 years |
| Human studies published in English between 1 January 2010 and 31 December 2025 | Non-MS neurological populations (e.g., spinal cord injury, Parkinson’s disease, stroke) unless MS data reported separately |
| Reported neurogenic bladder (NGB) or lower urinary tract dysfunction (LUTD) in PwMS (primary outcome or baseline/comorbidity variable) | Urinary dysfunction not attributable to MS or no extractable urinary outcome data |
| Reported or allowed calculation of prevalence, frequency, proportion, or incidence of NGB/LUTD | Did not report or allow calculation of NGB/LUTD prevalence or frequency |
| Observational study designs (cross-sectional, cohort, registry-based, or other epidemiologic designs) | Case reports, case series, reviews, editorials, letters, dissertations, conference abstracts |
| Probability-based, representative, or registry sampling methods | Interventional or experimental trials |
| Most recent or most complete dataset included when overlapping cohorts existed | Purposive or convenience sampling (unless part of representative registry/cohort) |
|  | Sample size < 30 |
|  | Duplicate datasets without additional unique data |
|  | Animal studies, in vitro studies, or non-English publications |

**Supplementary table 1: JBI Critical Appraisal Checklist**

| **Study** | **Q1** | **Q2** | **Q3** | **Q4** | **Q5** | **Q6** | **Q7** | **Q8** | **Q9** | **Overall** |
| --- | --- | --- | --- | --- | --- | --- | --- | --- | --- | --- |
| **Zecca 2016** | Low | Low | Low | Low | Low | Low | Low | Low | Low | Low |
| **Ronning 2016** | Low | Low | Low | Low | Low | Low | Low | Low | Low | Low |
| **Khalaf 2016** | Low | Low | Low | Low | **Moderate** | **Moderate** | **Moderate** | Low | **Moderate** | **Moderate** |
| **Tepavcevic 2016** | Low | Low | Low | Low | Low | Low | Low | Low | Low | Low |
| **Lin 2019** | Low | Low | Low | Low | **Moderate** | **Moderate** | **Moderate** | Low | **Moderate** | **Moderate** |
| **Tome 2019** | Low | Low | Unclear | Low | **Moderate** | **Moderate** | Low | Low | **Moderate** | **Moderate** |
| **Cabreira 2020** | Low | Low | Low | Low | **Moderate** | **Moderate** | **Moderate** | Low | **Moderate** | **Moderate** |
| **Nazari 2020** | Low | Low | Low | Low | Low | Low | Low | Low | Low | Low |
| **Castelo-Branco 2020** | Low | Low | Low | Low | **Moderate** | **Moderate** | **Moderate** | Low | **Moderate** | **Moderate** |
| **Azadvari 2020** | Low | Low | Low | Low | Low | Low | Low | Low | Low | Low |
| **Kapica-Topczewska 2020** | Low | Low | Low | Low | **Moderate** | Low | Low | Low | **Moderate** | Low |
| **Seddone 2021** | Low | Low | Low | Low | Low | Low | Low | Low | Low | Low |
| **Jacob 2021** | Low | Low | Low | Low | **Moderate** | **Moderate** | Low | Low | **Moderate** | **Moderate** |
| **Carotenuto 2021** | Low | Low | Low | Low | **Moderate** | **Moderate** | Low | Low | **Moderate** | Low |
| **Ferreira Resstel 2023** | Low | Low | Unclear | Low | **Moderate** | **Moderate** | **Moderate** | Low | **Moderate** | **Moderate** |
| **El Moudane 2023** | Low | Low | Low | Low | **Moderate** | **Moderate** | **Moderate** | Low | **Moderate** | **Moderate** |
| **Sharifiaghdas 2023** | Low | Low | Low | Low | Low | Low | Low | Low | Low | Low |
| **Luzanova 2024** | Low | Low | Unclear | Low | Low | Low | Low | Low | Low | Low |
| **Balsamo 2024** | Low | Low | Low | Low | Low | Low | Low | Low | Low | Low |
| **Rzepka 2024** | Low | Low | Low | Low | Low | Low | Low | Low | Low | Low |
| **Said 2025** | Low | Low | Low | Low | **Moderate** | **Moderate** | **Moderate** | Low | **Moderate** | **Moderate** |
| **Krhut 2025** | Low | Low | Low | Low | **Moderate** | Low | Low | Low | **Moderate** | **Moderate** |

**Supplementary table 2 : Leave one out analysis (GLMM)**

| **Study** | **Pooled Proportion (Logit)** |
| --- | --- |
| Zecca et al 2016 | -0.019609 |
| Rønning et al 2016 | 0.009494 |
| Khalaf et al 2016 | -0.150014 |
| Tepavcevic et al.2016 | -0.066179 |
| Lin et al.20219 | -0.109221 |
| Tomé et al. 2019 | -0.189060 |
| Cabreira et al 2020 | -0.011150 |
| Nazari et al. 2020 | -0.102190 |
| Castelo-Branco et al. 2020 | 0.086807 |
| Azadvari et al.2020 | -0.003962 |
| Kapica-Topczewska et al. 2020 | -0.027849 |
| Seddone et al 2021 | -0.054630 |
| Jacob et al.2021 | 0.046491 |
| Carotenuto et al 2021 | -0.029455 |
| Ferreira Resstel et al 2023 | -0.046552 |
| El Moudane et al 2023 | -0.112329 |
| Sharifiaghdas et al 2023 | -0.101793 |
| Luzanova et al 2024 | -0.063440 |
| Balsamo et al 2024 | 0.024528 |
| Rzepka et al 2024 | -0.088771 |
| Said et al 2025 | 0.024371 |
| Khurt et al 2025 | -0.070566 |

**Supplementary table 3: Prediction interval analysis**

| **Statistic** | **Lower Bound** | **Upper Bound** | **Scale** |
| --- | --- | --- | --- |
| Prediction Interval | -2.77 | 2.67 | Logit |
| Prediction Interval | 0.059 | 0.935 | Proportion |
| Prediction Interval | 5.9% | 93.5% | Percentage |

**Supplementary table 4: Trim and fill results**

| **k (Total Studies)** | **Added Studies** | **Pooled Proportion** | **95% Confidence Interval** | **I²** |
| --- | --- | --- | --- | --- |
| 32 | 10 | 0.2077 | 0.1008 , -0.3800 | 99.7% |

**Supplementary figure 1: Robins-E Traffic light plot**

**Supplementary figure 2: Funnel plot**
